# Safety and effectiveness of Salvia miltiorrhiza and ligustrazine injection for acute cerebral infarction in Chinese population: a PRISMA-compliant meta-analysis

**DOI:** 10.3389/fphar.2024.1425053

**Published:** 2024-12-02

**Authors:** Zhuoya Ma, Hu Zhang, Fen Zhao, Ke Li, Nanhai Dong, Wenwen Sang

**Affiliations:** ^1^ Department of Neurology, Liaocheng People’s Hospital, Liaocheng, Shandong, China; ^2^ Department of Emergency Medicine, Taizhou People’s Hospital, Taizhou, Jiangsu, China; ^3^ Department of Clinical Laboratory, Fushan District People’s Hospital of Yantai City, Yantai, Shandong, China; ^4^ Department of Central Laboratory, Liaocheng People’s Hospital, Liaocheng, Shandong, China; ^5^ Department of Clinical Laboratory, Liaocheng People’s Hospital, Liaocheng, Shandong, China

**Keywords:** Salvia miltiorrhiza and ligustrazine injection, traditional Chinese herbal medicine injection, acute cerebral infarction, regular treatments, meta-analysis

## Abstract

**Background:**

Salvia miltiorrhiza and ligustrazine injection (SML) is a type of traditional Chinese medicine injection, which has been considered a promising adjunctive therapy treatment for acute cerebral infarction (ACI). Although there have been positive reports on the treatment of SML, there is still controversy over its exact efficacy and safety in ACI patients. In this study, a systematic review was conducted on randomized controlled trials (RCTs) of SML for the treatment of ACI to evaluate its clinical efficacy and safety.

**Method:**

From the establishment of the database until May 2023, all randomized controlled trials related to SML and ACI were collected from the Cochrane Library, Web of Science, Embase, Medline, PubMed, CSJD, Wanfang database, CBM and CNKI. This systematic review and meta-analysis were strictly conducted in accordance with the PRISMA statement. The reported outcomes including overall response (ORR), National Institutes of Health Stroke Scale (NIHSS), hemorrheology indexes, activity of daily living (ADL) and adverse events were in detail investigated.

**Results:**

An analysis was conducted on the relevant data of 3869 ACI patients from 38 trials. The results indicated that the combination of conventional treatment and SML can significantly improve the ORR of patients (RR = 1.23, 95% CI = 1.20–1.27, *P* < 0.00001), neurological status (NIHSS, MD = −4.35, 95% CI = −5.15–3.54, *P* < 0.00001) and ADL (Barthel Index score, MD = 10.27, 95% CI = 7.75–12.79, *P* < 0.00001) compared with regular treatment alone. After the combined therapy, the hemorheology of ACI patients also significantly improved (*P* < 0.05). There is no significant difference in the frequency of adverse events between the two groups (RR = 1.49, 95% CI = 0.91–2.46, *P* = 0.11).

**Conclusion:**

The evidence from the meta-analysis suggested that the combination of conventional therapy and SML is safer and more effective than conventional therapy alone in treating ACI. However, due to the limitations of this analysis, such as regional bias and publication bias, the above conclusions need to be further verified by prospective, high-quality and multicenter clinical trials.

## 1 Introduction

Acute cerebral infarction (ACI) is a common cardiovascular disease caused by partial or extensive cerebral vascular stenosis or occlusion, which can lead to an impaired blood flow to the brain, followed by neurological damage and cerebral ischemia, even death (Liu Y. et al., 2019; Powers et al., 2019; Ma et al., 2017). There are more than 50 million people suffering from cerebral infarction worldwide, and nearly half of stroke survivors leave sequelae (Xue et al., 2019). The annual growth rate of the incidence rate of stroke is 8.7% in China, which is the main cause of death of Chinese people and exceeds the world average (Zhou et al., 2020; Wang W. et al., 2017). The proportion of ACI in stroke cases is 69.6%–70.8%, with a prevalence rate of 2.19%, and an annual death toll of ACI is about 1.96 per million (Li et al., 2020; Wang D. et al., 2017). In addition to seriously affecting the quality of life of patients, it also brings great psychological and economic burden to patients and their families (Zhou et al., 2020; Zeng et al., 2005). In the ultra-early stage of cerebral infarction, thrombolytic therapy is one of the most important method for restoring blood flow, which can improve blood circulation in and around the infarct area to avoid or reduce secondary nerve damage (Powers et al., 2019; Zhou et al., 2020). However, a strict time window determines the success or failure of thrombolytic therapy, and it is estimated that only less than 3% of patients have benefited from the treatment (Li et al., 2020; Lyu et al., 2019; Liu H. et al., 2019; Asadi et al., 2015). Reasonable application of antiplatelet drugs and nutritional therapy regimens can contribute to improve symptoms, but may increase the possibility of intracranial hemorrhage (Powers et al., 2019; Li et al., 2020). Therefore, finding an safe and effective adjuvant drug therapy for ACI is urgently needed in clinical practice.

Salvia miltiorrhiza and ligustrazine injection (SML) is a phytochemical agent that synthesized by tanshinol, which is extracted from Danshen (Radix Salviae Miltiorrhiae), and Ligustrazine Hydrochloride, which is extracted from Chuanqiong (Rhizoma Chuanqiong) (Ye et al., 2021; Zhang et al., 2016). Researches have proved that tanshinol can increase coronary blood flow, improve coronary microcirculation and hemorheology indexes, and reduce the degree of cerebral ischemia (Zhang et al., 2016; Deng et al., 2021; Meim et al., 2019). As the main active ingredient of Chuanqiong, Ligustrazine can inhibit platelet aggregation and fibrosis, promotes vasodilation and eliminates blood stasis (Zhang et al., 2016; Deng et al., 2021; Ding et al., 2019; Gao et al., 2015). SML has the pharmacological characteristics of both, and has been used as a supplementary drug in treating ischemic cardiovascular and cerebrovascular diseases in numerous hospitals of China (Ye et al., 2021; Zhang et al., 2016; Deng et al., 2021; Huang W. et al., 2016). The network Meta-analysis by Peng et al. showed combination therapy of SML and conventional treatment can improve the comprehensive efficacy in treating ACI, reduce National Institutes of Health stroke scale (NIHSS) scores and Fibrinogen (FIB) level in the blood, and improve Barthel index levels (Peng et al., 2023). Compared to other traditional Chinese medicine preparations, the combination of SML and western medicine had the best effect on reducing platelet aggregation rate in the blood (Peng et al., 2023). Previous studies have demonstrated the safety and efficacy of SML in treating cardiovascular and cerebrovascular diseases. However, most of the previous studies only evaluated a small number of indicators, and could not comprehensively evaluate the clinical efficacy of SML in treating ACI.

**FIGURE 1:**
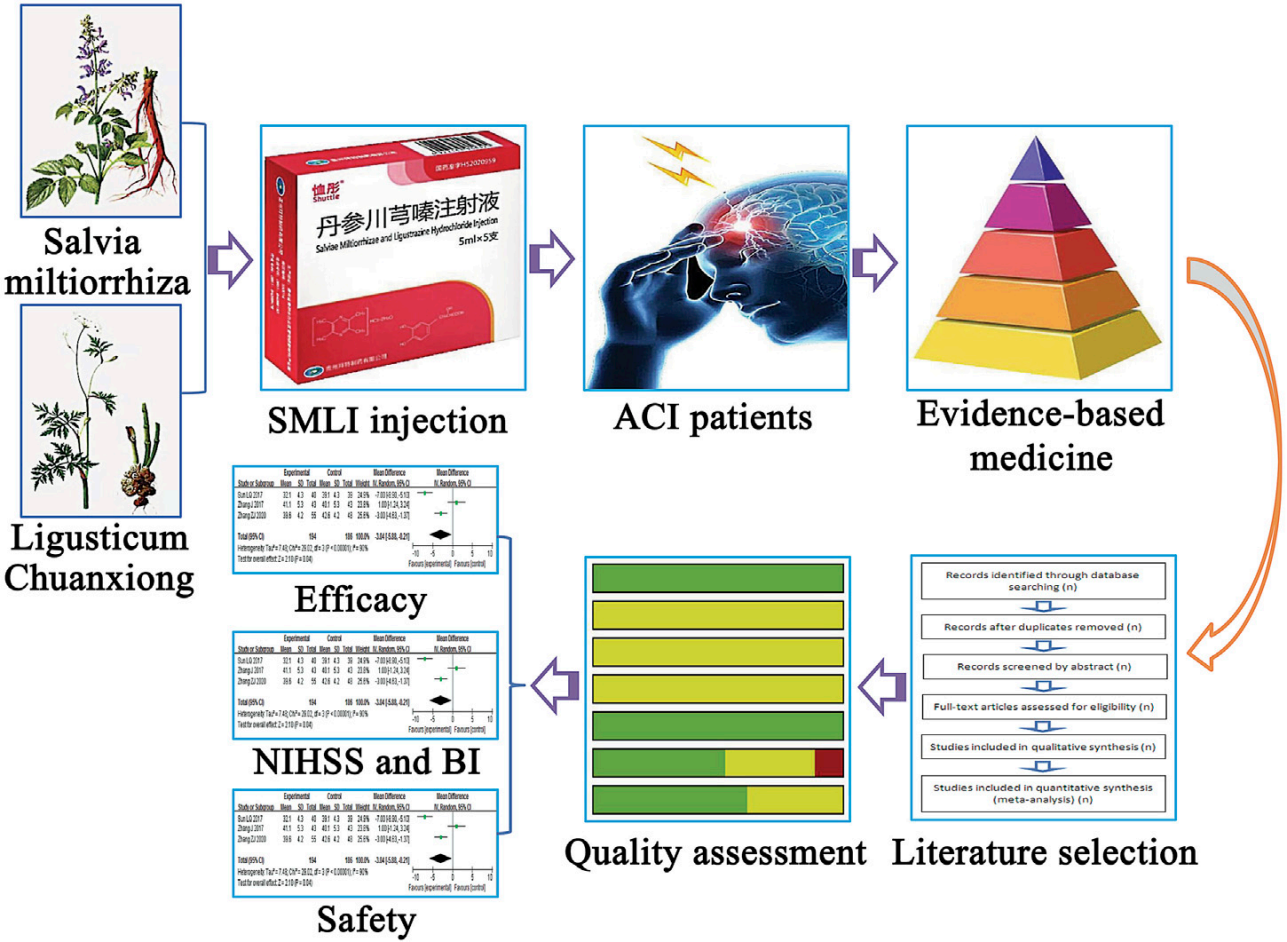
Work flow of the present study. SMLI, Salvia miltiorrhiza and ligustrazine injection; ACI, Aute cerebral infarction; NIHSS, National Institutes of Health Stroke Scale; BI, Barthel Index.

The present study conducted a systematic meta-analysis to fully evaluate the safety and efficacy of the combination of conventional therapy and SML for the treatment of ACI, compared to regular treatment alone, in order to provide scientific references for the design and implementation of future clinical trials (Figure 1. Work flow of the present study).

## 2 Methods

This meta-analysis was conducted in accordance with the Preferred Reporting Items for Systematic Reviews and Meta-Analyses (PRISMA) guidelines (Moher et al., 2009). As this project does not require recruiting patients or collecting personal information, further ethical approval is not required. The data were independently extracted from relevant literatures by two researchers, which are completely anonymous with no personal information being collected. All the information collected will be kept confidential and not disclosed to the public. It is necessary for researchers to ensure the privacy of patients while maintaining the accuracy and completeness of the collected data during the data extraction process.

### 2.1 Search strategy

Relevant Literatures were searched in nine electronic databases, including Cochrane Library, Web of Science, Embase, Medline, PubMed, Wanfang database, Chinese Scientific Journal Database (VIP), Chinese Biological Medicine Database (CBM) and China National Knowledge Infrastructure (CNKI). Chinese and English publications from the establishment of the database until May 2023 were shortlisted using the following search terms: “Danshen Chuanqiongqin” or “Danshen Chuanqiongqin injection” or “Salviae Miltiorrhizae and Ligustrazine” or “Radix Salivae Miltiorrhizae Ligustrazine injection” or “Salvia Miltiorrhiza Ligustrazine injection” or “Salviae Miltiorrhizae and Ligustrazine Hydrochloride injection” combined with “cerebral infarction” or “acute cerebral infarction” or “brain infarction” or “infarction of the brain” or “ischemic stroke” or “ACI”. No other restrictive search criteria were applied (Supplementary Table S1).

### 2.2 Eligibility criteria

• Inclusion criteria
  (I) Randomized controlled trials (RCTs) involving ACI patients were included.
  (II) The study subjects (ACI patients) must meet the diagnostic criteria of the World Health Organization for ACI and rule out cerebral hemorrhage through magnetic resonance imaging (MRI) or computed tomography (CT).
  (III) Articles involving more than 40 ACI patients.
  (IV) Literatures comparing the clinical outcomes of conventional treatment with SML adjuvant therapy (experimental group) and conventional single therapy (control group).
  (V) Overall response rate (ORR) and NIHSS must be included in every study.
• Exclusion criteria
  (I) Unrelated SML studies were excluded.
  (II) Inappropriate criteria were excluded in control or experimental group.
  (III) Articles with insufficient available data were excluded.
  (IV) Non randomized controlled trials, literature reviews, meta-analysis, conference abstracts, repeated studies, case reports, and experimental model researches were excluded.

### 2.3 Quality assessment

To ensure the quality of the meta-analysis, the quality of the included randomized controlled trials was assessed using the Cochrane Handbook tool (Zeng et al., 2015). The assessment tool includes the following seven items: (I) random sequence generation, (II) allocation concealment, (III) blinding of participants and personnel, (IV) blinding of outcome assessment, (V) incomplete outcome data, (VI) selective reporting and (VII) other bias. Each item is divided into three levels: low risk, unclear and high risk.

### 2.4 Types of outcome measures

• Main outcomes
  (I) ORR
  (II) NIHSS
  (III) BI score.
• Secondary outcomes
  (I) Hemorrheology indexes: Whole blood viscosity (WBV), Plasma viscosity (PV), Whole blood low-shear viscosity (WBLSV), Whole blood high-shear viscosity (WBHSV), FIB, Platelet aggregation rate (PAR) and Hematocrit (HCT).
  (II) The level of C-reactive protein (CRP).
  (III) Treatment-related adverse events (TRAE).

### 2.5 Data extraction and management

The data were independently extracted by two researchers (ZYM and HZ) using the inclusion and exclusion criteria mentioned above; The disagreements will be adjudicated by the third reviewer (FZ).

The data were taken from eligible studies:

• Research characteristics, such as first author name, patient ages, publication time, number of cases, and study parameter types.
• Details of the intervention measures, such as intervention techniques, dosage, route of administration, manufacturer information of SML, and duration of SML treatment.
• Outcomes measures and other indicators, such as the ORR, NIHSS, BI, Hemorrheology indexes, CRP, and TRAE.

Missing or incomplete data will be obtained by contacting the authors. If the relevant data could not be obtained, these studies were excluded from the analysis.

### 2.6 Statistical analysis

The statistical analyses were conducted using Review Manager 5.3 (Nordic Cochran Centre, Copenhagen, Denmark) and Stata 14.0 (Stata Corp., College Station, TX, United States). Dichotomous data is represented by risk ratio (RR) and their respective 95% confidence intervals (CI), while continuous variables are represented by mean difference (MD) and 95% confidence intervals. *P* < 0.05 indicates that the difference is statistically significant. Heterogeneity between studies was evaluated using the Cochran’s Q statistic and *I* ^*2*^ tests, and *I* ^*2*^ > 50% or *P* < 0.1 indicated a high level of statistical heterogeneity (Jackson et al., 2012). When there is no heterogeneity (*I* ^*2*^ < 50%), a fixed-effects model was used to pool the estimates. Otherwise, a random effects model was chosen.

Any publication bias survey was conducted on the parameters reported in more than 10 studies using funnel plots and Begg and Egger tests (Lin and Chu, 2018; Begg and Mazumdar, 1994; Egger et al., 1997). If there is publication bias, a trim-and-fill method was are used to reconcile the estimates of unpublished studies, and the adjusted results were compared with the original pooled RR (Duval and Tweedie, 2000). To investigate the impact of treatment duration and SML manufacturer on clinical efficacy, subgroup analysis was conducted.

## 3 Results

### 3.1 Search results

**FIGURE 2:**
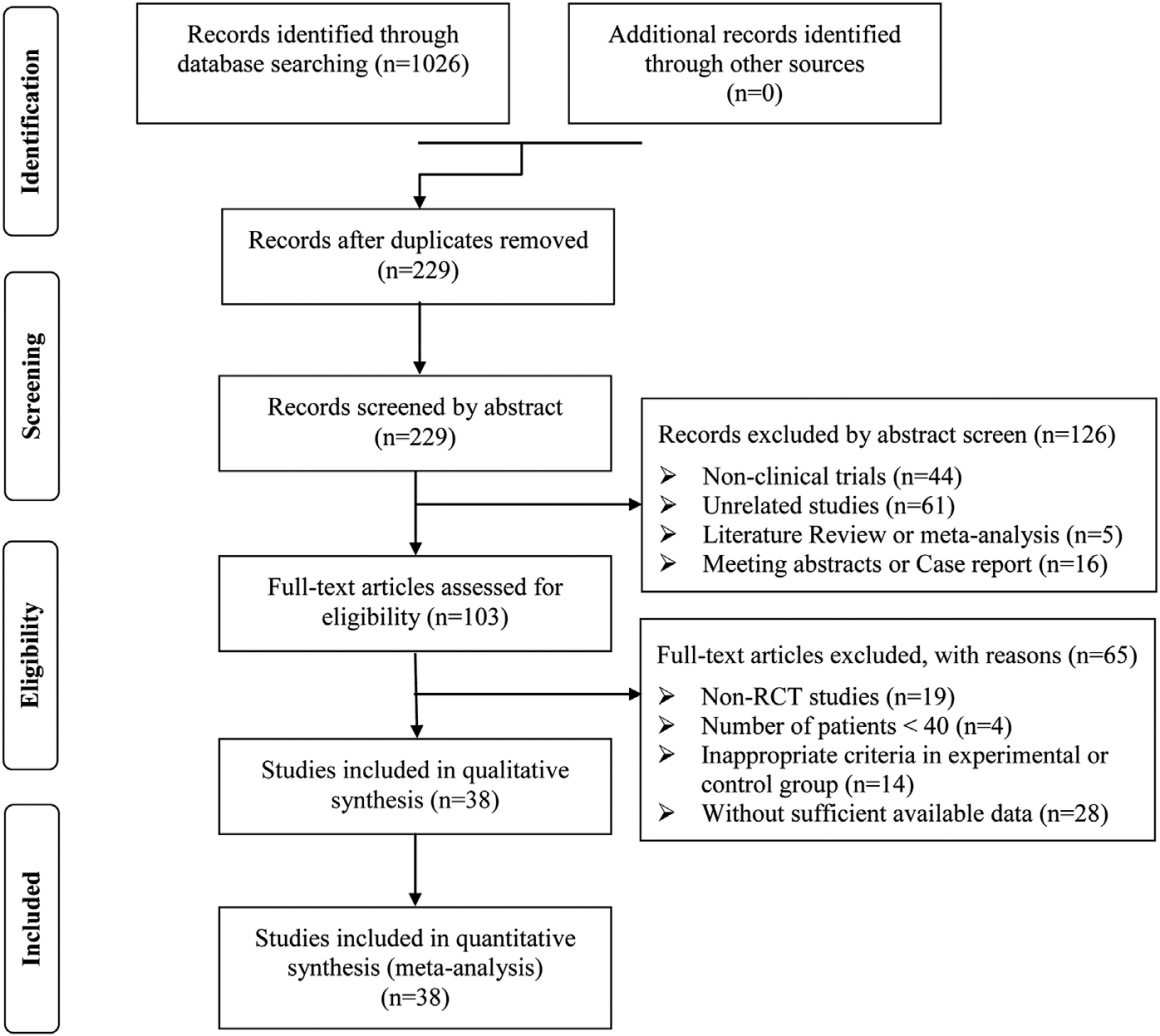
Study selection process for the meta-analysis.

After preliminary search, a total of 1,026 articles were identified. 797 papers were excluded from the study due to duplication issues. After reviewing the titles and abstracts, 229 articles were further excluded as they were not unrelated studies (n = 61) or clinical trials (n = 44) or literature review and meta-analysis (n = 5) or case report and meeting abstract (n = 16), and the remaining 103 studies have potential relevance. After a detailed evaluation of the entire text, articles were not RCTs (n = 19), studies with a sample size of less than 40 (n = 4). This study excluded publications with inappropriate standards for the experimental or control group (n = 14) and trials with insufficient data (n = 28). Finally, this analysis included 38 trials (Dai and Wu, 2018; Guo and Guan, 2018; Guo, 2015; Han, 2021; Huang SG. et al., 2016; Ji, 2022; Jiang and Xia, 2019; Lan et al., 2015; Li CL. et al., 2016; Li DQ., 2018; Li, 2021; Li L., 2018; Li and Liu, 2017; Li et al., 2017; Li TD. et al., 2016; Li ZL. et al., 2016; Liu, 2017; Liu, 2018; Liu, 2014; Mamuti, 2017; Qu and Liu, 2016; Song, 2018; Sun, 2017; Tan, 2016; Tan, 2019; Wan et al., 2015; Wang and Wu, 2018; Wang and Zhang, 2016; Xu B. et al., 2021; Xu, 2017; Xu Z. et al., 2021; Yan, 2016; Yang et al., 2010; Yu et al., 2019; Zhang and Li, 2013; Zhang, 2019; Zhang and Xiao, 2020; Zhao and Zhou, 2020) involving 3869 ACI patients (Figure 2).

### 3.2 Patient characteristics

After screening, all included trials were conducted in different medical centers in China. The information of the hospitals conducting the relevant research was showed in Supplementary Table S3. A total of 1,962 ACI patients received conventional treatment combined with SML adjuvant therapy, and 1,907 patients received conventional treatment alone. The detailed information on relevant studies and ACI patients is shown in Table 1. Except for one trial (Li and Liu, 2017), all included trials clearly introduced the duration of SML treatment. Twenty-seven studies (Dai and Wu, 2018; Guo and Guan, 2018; Guo, 2015; Han, 2021; Ji, 2022; Jiang and Xia, 2019; Lan et al., 2015; Li CL. et al., 2016; Li DQ., 2018; Li, 2021; Li L., 2018; Li and Liu, 2017; Li et al., 2017; Li TD. et al., 2016; Li ZL. et al., 2016; Liu, 2014; Qu and Liu, 2016; Song, 2018; Sun, 2017; Tan, 2016; Tan, 2019; Wang and Wu, 2018; Xu B. et al., 2021; Xu, 2017; Xu Z. et al., 2021; Yang et al., 2010; Zhang and Li, 2013; Zhang and Xiao, 2020; Zhao and Zhou, 2020) specifically describe the manufacturer of SML and the other nine studies (Huang SG. et al., 2016; Liu, 2017; Liu, 2018; Mamuti, 2017; Wan et al., 2015; Wang and Zhang, 2016; Yan, 2016; Yu et al., 2019; Zhang, 2019) lacked clear description of production information (Table 1). The SML quality standards used in this study have been approved by Chinese State Food and Drug Administration (SFDA), and have obtained the corresponding production approval number (H52020959 or H22026448). All relevant pharmaceutical companies followed the quality handling procedures specified in the pharmacopoeia.

**TABLE 1:** Clinical information from the eligible trials in the meta-analysis.

Included studies	Patients Con/Exp	Age (year)		Intervening measure (Exp vs. Con)	Dosage of SMLI	Duration of treatment	Parameter types
		Con	Exp				
Dai XK 2018	42/42	61.62 ± 3.64 (mean)	61.74 ± 3.56 (mean)	RT + SMLI (iv)^a^ vs. RT	10 mL/Day	1 month	ORR, NIHSS
Guo J 2018	59/59	59.54 ± 11.31 (mean)	60.33 ± 12.18 (mean)	RT + SMLI (iv)^b^ vs. RT	10 mL/Day	2 weeks	ORR, NIHSS, BI, HI
Guo Y 2015	48/48	63.8 ± 10.6 (mean)	62.5 ± 11.2 (mean)	RT + SMLI (iv)^b^ vs. RT	10 mL/Day	2 weeks	ORR, NIHSS, ADE
Han SP 2021	21/21	62.34 ± 3.52 (mean)	62.18 ± 3.50 (mean)	RT + SMLI (iv)^a^ vs. RT	5 mL/Day	4 weeks	ORR, NIHSS, BI, HI
Huang SG 2016	39/39	Not provided	Not provided	RT + SMLI (iv)^c^ vs. RT	10 mL/Day	15 days	ORR, NIHSS, ADE
Ji DY 2022	48/48	51.36 ± 4.11 (mean)	52.45 ± 4.16 (mean)	RT + SMLI (iv)^a^ vs. RT	10 mL/Day	2 weeks	ORR, NIHSS, BI, CRP, ADE
Jiang KY 2019	36/36	Not provided	Not provided	RT + SMLI (iv)^c^ vs. RT	10 mL/Day	15 days	ORR, NIHSS
Lan Y 2015	40/40	56–70 (range)	54–72 (range)	RT + SMLI (iv)^c^ vs. RT	10 mL/Day	2 weeks	ORR, NIHSS, BI, ADE
Li CL 2016	84/84	67.2 ± 8.8 (mean)	68.1 ± 8.7 (mean)	RT + SMLI (iv)^b^ vs. RT	10 mL/Day	2 weeks	ORR, NIHSS, BI
Li DQ 2018	40/40	67.29 ± 6.23 (mean)	68.01 ± 6.46 (mean)	RT + SMLI (iv)^a^ vs. RT	5 mL/Day	2 weeks	ORR, NIHSS
Li h 2021	47/47	64.30 ± 5.16 (mean)	65.48 ± 5.33 (mean)	RT + SMLI (iv)^a^ vs. RT	5 mL/Day	2 weeks	ORR, NIHSS, CRP, HI, ADE
Li L 2018	36/36	61.5 ± 4.1 (mean)	61.2 ± 4.5 (mean)	RT + SMLI (iv)^a^ vs. RT	10 mL/Day	2 weeks	ORR, NIHSS, HI
Li SH 2017	45/45	78.3 ± 4.9 (mean)	75.1 ± 3.2 (mean)	RT + SMLI (iv)^b^ vs. RT	10 mL/Day	Not provided	ORR, NIHSS, BI
Li T 2017	98/105	59.5 ± 14.1 (mean)	60.1 ± 16.0 (mean)	RT + SMLI (iv)^b^ vs. RT	10 mL/Day	2 weeks	ORR, NIHSS, BI, HI
Li TD 2016	41/41	50.2 ± 2.6 (mean)	50.6 ± 2.4 (mean)	RT + SMLI (iv)^b^ vs. RT	10 mL/Day	15 days	ORR, NIHSS
Li ZL 2016	40/40	53.12 ± 5.32 (mean)	53.28 ± 5.17 (mean)	RT + SMLI (iv)^a^ vs. RT	10 mL/Day	4 weeks	ORR, NIHSS, HI, ADE
Liu H 2017	40/40	64.3 ± 3.8 (mean)	58.1 ± 3.2 (mean)	RT + SMLI (iv)^c^ vs. RT	10 mL/Day	10 days	ORR, NIHSS
Liu JX 2018	50/50	43.58 ± 11.50 (mean)	44.58 ± 10.50 (mean)	RT + SMLI (iv)^c^ vs. RT	10 mL/Day	2 weeks	ORR, NIHSS
Liu M 2014	68/68	60.4 ± 16.3 (mean)	59.8 ± 15.7 (mean)	RT + SMLI (iv)^b^ vs. RT	10 mL/Day	2 weeks	ORR, NIHSS, ADE
Mamuti A 2017	38/38	62.4 ± 6.8 (mean)	61.8 ± 7.1 (mean)	RT + SMLI (iv)^c^ vs. RT	10 mL/Day	2 weeks	ORR, NIHSS, BI
Qu J 2016	75/75	62.35 ± 11.46 (mean)	61.52 ± 12.38 (mean)	RT + SMLI (iv)^b^ vs. RT	10 mL/Day	2 weeks	ORR, NIHSS, BI
Song HY 2018	48/48	61 ± 11 (mean)	61 ± 11 (mean)	RT + SMLI (iv)^b^ vs. RT	20 mL/Day	2 weeks	ORR, NIHSS, ADE
Sun LQ 2017	39/40	51.6 ± 4.6 (mean)	51.4 ± 4.8 (mean)	RT + SMLI (iv)^a^ vs. RT	5 mL/Day	1 week	ORR, NIHSS, BI, HI, ADE
Tan GL 2016	56/56	68.8 ± 4.3 (mean)	68.6 ± 4.2 (mean)	RT + SMLI (iv)^a^ vs. RT	10 mL/Day	2 weeks	ORR, NIHSS, BI, ADE
Tan HY 2019	50/50	65.8 ± 2.5 (mean)	65.5 ± 2.4 (mean)	RT + SMLI (iv)^b^ vs. RT	10 mL/Day	1 week	ORR, NIHSS, BI
Wan J 2015	41/41	59.2 ± 6.3 (mean)	61.4 ± 5.8 (mean)	RT + SMLI (iv)^c^ vs. RT	10 mL/Day	4 weeks	ORR, NIHSS, BI
Wang LN 2018	60/60	62.63 ± 7.79 (mean)	62.51 ± 7.86 (mean)	RT + SMLI (iv)^b^ vs. RT	5 mL/Day	2 weeks	ORR, NIHSS, ADE
Wang XM 2016	56/56	61.2 ± 5.9 (mean)	60.4 ± 5.7 (mean)	RT + SMLI (iv)^c^ vs. RT	10 mL/Day	2 weeks	ORR, NIHSS, BI, ADE
Xu B 2021	30/30	61.59 ± 7.04 (mean)	61.85 ± 7.23 (mean)	RT + SMLI (iv)^a^ vs. RT	10 mL/Day	4 weeks	ORR, NIHSS, BI, CRP
Xu HJ 2017	43/43	61.17 ± 5.23 (mean)	60.54 ± 5.46 (mean)	RT + SMLI (iv)^a^ vs. RT	5–10 mL/Day	2 weeks	ORR, NIHSS
Xu Z 2021	100/100	63.72 ± 8.43 (mean)	63.86 ± 7.59 (mean)	RT + SMLI (iv)^a^ vs. RT	10 mL/Day	2 weeks	ORR, NIHSS, BI, HI
Yan SJ 2016	30/30	54.2 ± 13.4 (mean)	51.3 ± 12.1 (mean)	RT + SMLI (iv)^c^ vs. RT	10 mL/Day	2 weeks	ORR, NIHSS, BI
Yang ZY 2010	31/31	55–65 (range)	50–65 (range)	RT + SMLI (iv)^b^ vs. RT	10 mL/Day	2 weeks	ORR, NIHSS
Yu J 2019	40/40	66.60 ± 10.38 (mean)	67.26 ± 10.96 (mean)	RT + SMLI (iv)^c^ vs. RT	10 mL/Day	1 week	ORR, NIHSS, BI
Zhang L 2013	56/56	55–76 (range)	56–75 (range)	RT + SMLI (iv)^b^ vs. RT	15 mL/Day	15 days	ORR, NIHSS
Zhang QY 2019	54/54	Not provided	Not provided	RT + SMLI (iv)^c^ vs. RT	10 mL/Day	2 weeks	ORR, NIHSS
Zhang ZJ 2020	88/135	58.0 ± 11.23 (mean)	56.0 ± 13.12 (mean)	RT + SMLI (iv)^b^ vs. RT	5 mL/Day	4 weeks	ORR, NIHSS, HI
Zhao JQ 2020	50/50	54.09 ± 3.28 (mean)	54.23 ± 3.25 (mean)	RT + SMLI (iv)^b^ vs. RT	10 mL/Day	4 weeks	ORR, NIHSS, ADE

Notes: Con, control group (regular treatments alone group); Exp, experimental group (regular treatments and SMLI, combined group).

^a^ Jilin Sichang Pharmaceutical Co., Ltd. (Manufacturing Approve Number, H22026448).

^b^ Guizhou Baite Pharmaceutical Co., Ltd. (Manufacturing Approve Number, H52020959).

^c^ Not provided.

Abbreviations: ORR: overall response rate; RT: regular treatments; SMLI: salvia miltiorrhiza and ligustrazine injection; iv: Intravenous drip; NIHSS: national institutes of health stroke scale; BI: barthel index score; HI: hemorrheology indexes; CPR; plasma C reactive protein; ADE: adverse events.

### 3.3 Quality assessment

**FIGURE 3:**
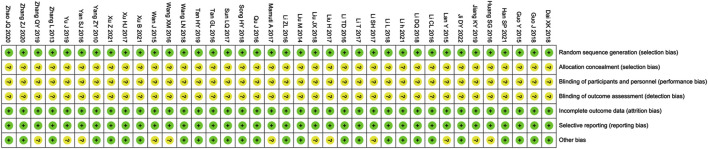
Risk of bias summary. Review of authors’ judgments about each risk of bias item for included studies. Note: Each color represents a different level of bias: red for high-risk, green for low-risk, and yellow for unclear-risk of bias.

The risk assessment of bias is shown in Figure 3. The selection, attrition and reporting risks of involved trials were low. There is no clear description provided by all included trials regarding performance and detection risks. Due to the lack of description of key information, Twelve trials (Huang SG. et al., 2016; Jiang and Xia, 2019; Lan et al., 2015; Li and Liu, 2017; Liu, 2017; Liu, 2018; Mamuti, 2017; Wan et al., 2015; Wang and Zhang, 2016; Yan, 2016; Yu et al., 2019; Zhang, 2019) were considered that the reported risks are unclear.

### 3.4 ORR assessments

**FIGURE 4:**
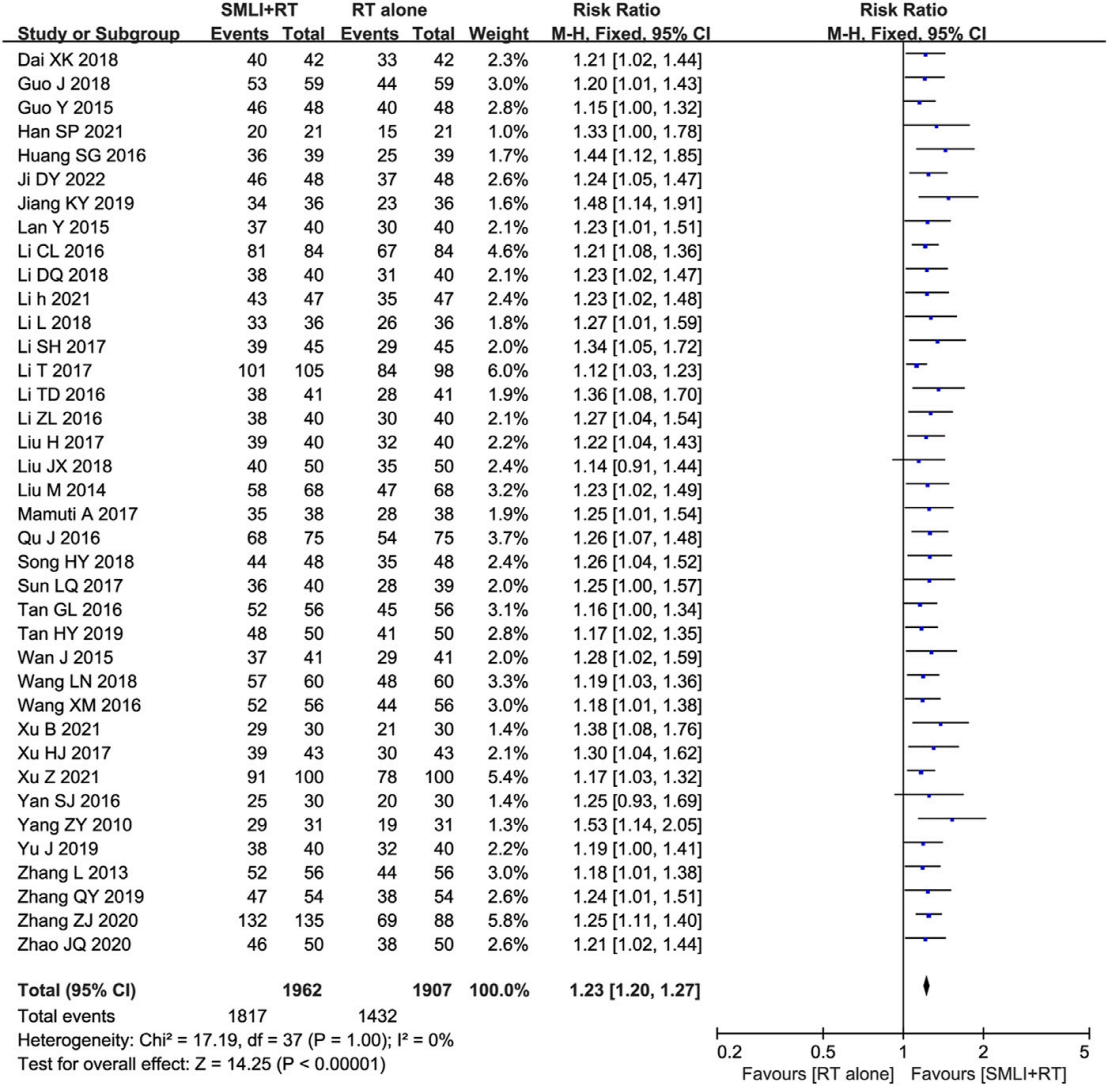
Forest plot of overall response rate in patients treated with RT + SMLT and RT alone. RT, Regular treatment; SMLI, Salvia miltiorrhiza and ligustrazine injection. The fixed effects meta-analysis model (Mantel-Haenszel method) was used.

Thirty-eight clinical trials (Dai and Wu, 2018; Guo and Guan, 2018; Guo, 2015; Han, 2021; Huang SG. et al., 2016; Ji, 2022; Jiang and Xia, 2019; Lan et al., 2015; Li CL. et al., 2016; Li DQ., 2018; Li, 2021; Li L., 2018; Li and Liu, 2017; Li et al., 2017; Li TD. et al., 2016; Li ZL. et al., 2016; Liu, 2017; Liu, 2018; Liu, 2014; Mamuti, 2017; Qu and Liu, 2016; Song, 2018; Sun, 2017; Tan, 2016; Tan, 2019; Wan et al., 2015; Wang and Wu, 2018; Wang and Zhang, 2016; Xu B. et al., 2021; Xu, 2017; Xu Z. et al., 2021; Yan, 2016; Yang et al., 2010; Yu et al., 2019; Zhang and Li, 2013; Zhang, 2019; Zhang and Xiao, 2020; Zhao and Zhou, 2020) involving 3,869 cases compared the ORR between the two groups (Figure 4). Our pooled results showed that compared to conventional treatments alone, patients receiving combined therapy had significantly improved ORR (RR = 1.23, 95% CI = 1.20–1.27, *P* < 0.00001). There was no heterogeneity, and a fixed-effect model was used for meta-analysis.

### 3.5 NIHSS

**FIGURE 5:**
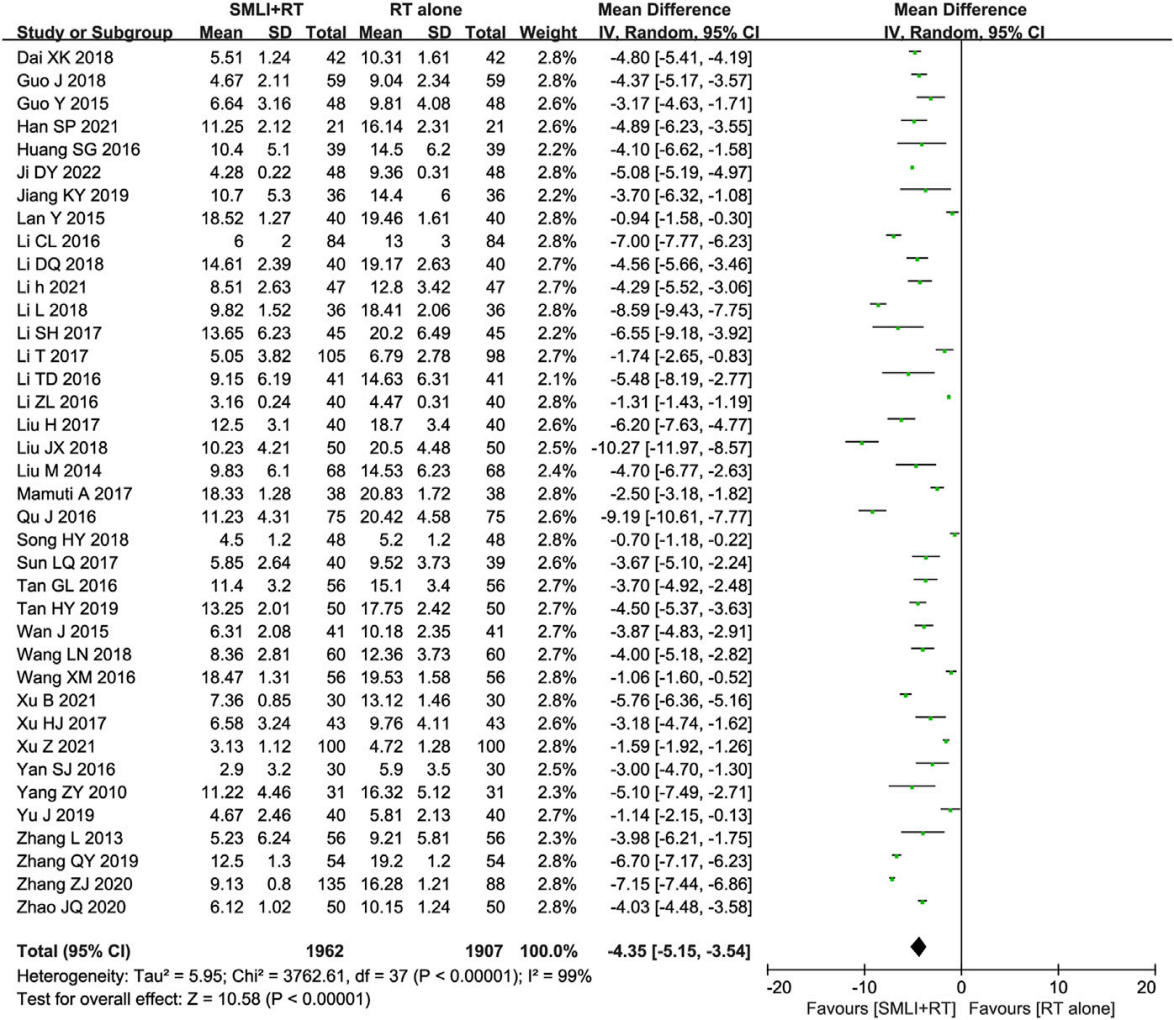
Forest plot of National Institutes of Health Stroke Scale in patients treated with RT + SMLT and RT alone. RT, Regular treatment; SMLI, Salvia miltiorrhiza and ligustrazine injection. The random effects meta-analysis model (Inverse Variance method) was used.

Thirty-eight trials (Dai and Wu, 2018; Guo and Guan, 2018; Guo, 2015; Han, 2021; Huang SG. et al., 2016; Ji, 2022; Jiang and Xia, 2019; Lan et al., 2015; Li CL. et al., 2016; Li DQ., 2018; Li, 2021; Li L., 2018; Li and Liu, 2017; Li et al., 2017; Li TD. et al., 2016; Li ZL. et al., 2016; Liu, 2017; Liu, 2018; Liu, 2014; Mamuti, 2017; Qu and Liu, 2016; Song, 2018; Sun, 2017; Tan, 2016; Tan, 2019; Wan et al., 2015; Wang and Wu, 2018; Wang and Zhang, 2016; Xu B. et al., 2021; Xu, 2017; Xu Z. et al., 2021; Yan, 2016; Yang et al., 2010; Yu et al., 2019; Zhang and Li, 2013; Zhang, 2019; Zhang and Xiao, 2020; Zhao and Zhou, 2020) with 3,869 participants measured their neurological status based on NIHSS (Figure 5). The results showed that compared to conventional treatment alone, ACI patients receiving combination therapy had significantly improved neurological status (MD = −4.35, 95% CI = −5.15–3.54, *P* < 0.00001). There was significant heterogeneity between studies (*I* ^*2*^ = 99%, *P* < 0.00001); Therefore, in order to pool data, we used a random effects model, so any conclusions need to be cautious.

### 3.6 BI score

**FIGURE 6:**
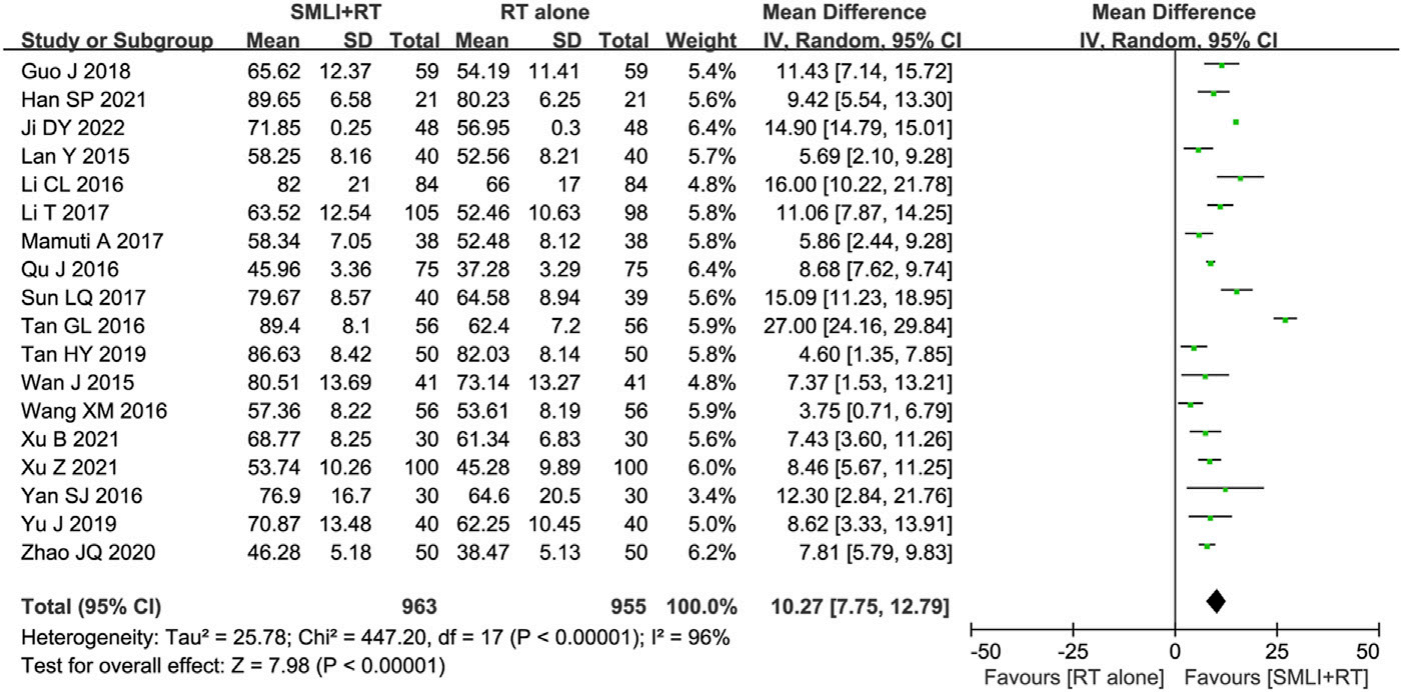
Forest plot of Barthel index score in patients treated with RT + SMLT and RT alone. RT, Regular treatment; SMLI, Salvia miltiorrhiza and ligustrazine injection. The random effects meta-analysis model (Inverse Variance method) was used.

Eighteen-trials (Guo and Guan, 2018; Han, 2021; Ji, 2022; Lan et al., 2015; Li CL. et al., 2016; Li et al., 2017; Mamuti, 2017; Qu and Liu, 2016; Sun, 2017; Tan, 2016; Tan, 2019; Wan et al., 2015; Wang and Zhang, 2016; Xu B. et al., 2021; Xu Z. et al., 2021; Yan, 2016; Yu et al., 2019; Zhao and Zhou, 2020) involving 1,918 ACI patient’s ability to perform daily living (ADL) was evaluated based on the BI score. As shown in Figure 6, the BI Score of ACI patients in the combined group were significantly higher than those in the control group (MD = 10.27, 95% CI = 7.75–12.79, *P* < 0.00001). A *P*-value <0.00001 and *I* ^*2*^ = 96% showed that there was significant heterogeneity between studies; Therefore, a random effect model was adopted.

### 3.7 CRP level

**FIGURE 7:**
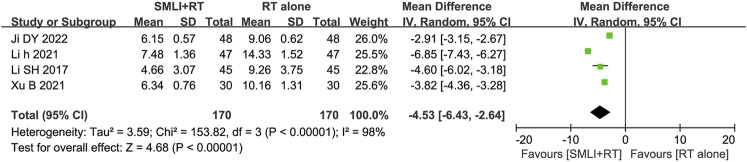
Forest plot of the serum C-reactive protein level in patients treated with RT + SMLT and RT alone. RT, Regular treatment; SMLI, Salvia miltiorrhiza and ligustrazine injection. The random effects meta-analysis model (Inverse Variance method) was used.

Four studies (Ji, 2022; Li, 2021; Li and Liu, 2017; Xu B. et al., 2021) involved 340 patients and measured CRP levels (Figure 7). The pooled analysis showed that compared with the conventional treatments, the combination of SML could significantly reduce the level of CRP in ACI patients (MD = −4.53, 95% CI = −6.43–2.64, *P* < 0.00001). According to heterogeneity testing, CRP level exhibits statistical heterogeneity (*P* < 0.00001, *I* ^*2*^ = 98%). Therefore, the random effects model was used for pooling this meta-analysis.

### 3.8 Hemorrheology assessment

Measurement of hemorheology in ACI patients was carried out between SML and non-SML groups in ten controlled studies (Guo and Guan, 2018; Han, 2021; Li, 2021; Li L., 2018; Li et al., 2017; Li ZL. et al., 2016; Sun, 2017; Tan, 2016; Xu Z. et al., 2021; Zhang and Xiao, 2020) (Supplementary Figures S1-S6). In this analysis, our results indicated that ACI patients receiving combination therapy have significantly improved hemorheology compared to those receiving conventional treatment alone, indicated by significantly reduced PV (MD = −0.40, 95% CI = −0.63–0.17, *P* = 0.0006), WBHSV (MD = −1.17, 95% CI = −1.44–0.89, *P* < 0.00001), WBLSV (MD = −1.47, 95% CI = −1.74–1.21, *P* < 0.00001), FIB (MD = −0.94, 95% CI = −1.62–0.26, *P* = 0.006), HCT (MD = −2.81, 95% CI = −5.50–0.11, *P* = 0.04) and PAR (MD = −10.46, 95% CI = −12.26–8.65, *P* < 0.00001). Given the high heterogeneity among studies, a random effects model was used to analyze their RR.

### 3.9 Adverse events assessment

**FIGURE 8:**
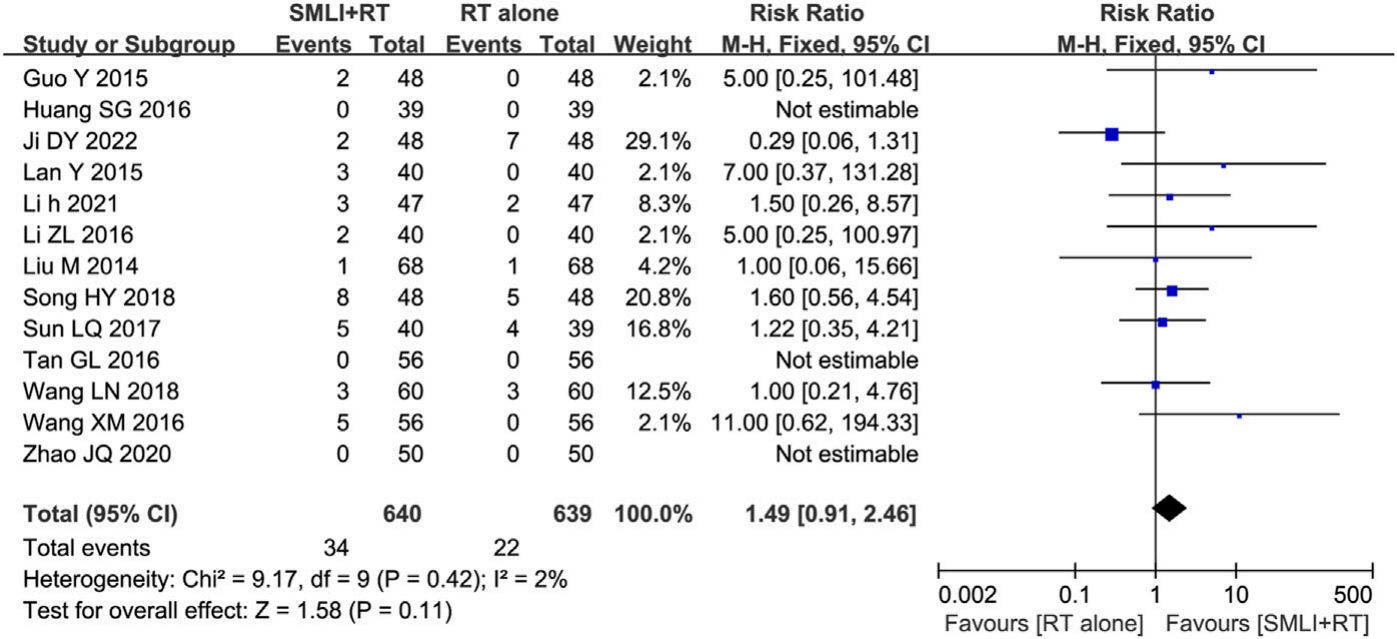
Forest plot of adverse effects in patients treated with RT + SMLT and RT alone. RT, Regular treatment; SMLI, Salvia miltiorrhiza and ligustrazine injection. The fixed effects meta-analysis model (Mantel-Haenszel method) was used.

Thirteen trials (Guo, 2015; Huang SG. et al., 2016; Ji, 2022; Lan et al., 2015; Li, 2021; Li ZL. et al., 2016; Liu, 2014; Song, 2018; Sun, 2017; Tan, 2016; Wang and Wu, 2018; Wang and Zhang, 2016; Zhao and Zhou, 2020) involving 1,279 ACI patients evaluated the safety of SML mediated therapy. Rash, headache, diarrhea, nausea, and vomiting are the most common side effects of SML treatment, and symptoms usually subside after treatment. There was no significant difference in the overall incidence of adverse events between the two groups (Figure 8, RR = 1.49, 95% CI = 0.91–2.46, *p* = 0.11). Due to low heterogeneity, fixed-effect models were used to analyze the RR rate.

### 3.10 Publication bias

Publication bias was assessed by Begg’s and Egger’s regression tests (Table 2), and was detected in indicators such as ORR, BI score and WBHSV. A trim-and-fill analysis was carried out to determine whether the publication bias affected the pooled risk. The adjusted RR showed the same trend as the preliminary analysis results (Table 2), reflecting the reliability of our preprimary conclusions.

**TABLE 2:** Summary of publication bias.

Publication bias	ORR	NIHSS	BI score	CRP	Hemorrheology indexes						Adverse events
					PV	WBHSV	WBLSV	FIB	HCT	PAR	
Begg	<0.0001	0.920	0.069	0.308	0.806	0.230	1.000	1.000	0.308	0.260	0.210
Egger	<0.0001	0.483	0.009	0.350	0.226	0.037	0.271	0.158	0.208	0.200	0.124
Trim and fill analysis											
before	*P* < 0.00001		*P* < 0.00001				*P* < 0.00001				
after	*P* < 0.00001		*P* < 0.00001				*P* < 0.00001				

Abbreviations: ORR: overall response rate; NIHSS: national institutes of health stroke scale; BI: barthel index; CRP: C-reactive protein; WBHSV: Whole blood high-shear viscosity; WBLSV: Whole blood low-shear viscosity; FIB: fibrinogen; HCT: hematocrit; PAR: platelet aggregation rate.

### 3.11 Sensitivity analysis

Sensitivity analysis was conducted to explore the impact of individual studies on the pooled results by removing a separate study from each pooled analysis. As shown in Supplementary Table S2, the results showed that no single study significantly affected the primary outcome measurement, indicating statistically robust results.

We also conducted subgroup analysis to explore the heterogeneity source of ORR and NIHSS in terms of treatment duration and SML manufacturers. As shown in Table 3, our analysis indicated that these variables have no significant impact on the efficacy of SML in treating ACI.

**TABLE 3:** Subgroup analyses of ORR and NIHSS between the experimental and control group.

Parameter	Factors at study level	Experimental group No. patients (n)	Control group No. patients (n)	Analysis method	Heterogeneity		Risk ratio (RR)	95% CI	*P*-value
					*I* ^*2*^ (%)	*P*-value			
ORR	Duration of treatment								
	1–2 weeks	1,386	1,378	Fixed	0	1.00	1.21	1.17–1.25	<0.00001
	>2 weeks	531	484	Fixed	0	0.90	1.28	1.21–1.36	<0.00001
	Manufacturer of the SMLI								
	I	955	901	Fixed	0	0.84	1.22	1.17–1.27	<0.00001
	II	543	542	Fixed	0	0.99	1.23	1.17–1.30	<0.00001
NIHSS	Duration of treatment								
	1–2 weeks	1,386	1,378	Random	98	<0.00001	−4.23	−5.13––3.32	<0.00001
	>2 weeks	531	484	Random	99	<0.00001	−4.46	−6.41––2.52	<0.00001
	Manufacturer of the SMLI								
	I	955	901	Random	98	<0.00001	−4.74	−6.17––3.32	<0.00001
	II	543	542	Random	100	<0.00001	−4.28	−5.71––2.86	<0.00001

Notes: Con, control group (regular treatments alone group); Exp, experimental group (regular treatments and SMLI, combined group). I: Guizhou Baite Pharmaceutical Co., ltd; II: Jilin Sichang Pharmaceutical Co., ltd.

Abbreviations: ORR: overall response rate; SMLI: salvia miltiorrhiza and ligustrazine injection; NIHSS: national institutes of health stroke scale.

### 3.12 Quality of evidence

There were 11 important outcomes in this meta-analysis, and the quality was low for ORR, BI, CRP and WBHSV; and moderate for other results (ADE, NIHSS, PV, WBHSV, WBLSV, FIB, HCT and PAR). Risk of bias and publication bias were the common reason for reducing the quality of evidence. Moreover, the total sample size is less than 400 cases for CRP, and their quality was downgraded by one level. No evidence was downgraded because of indirectness and inconsistency (see Table 4 in detail).

**TABLE 4:** GRADE evidence profile.

Indicators (RCTs)	Risk of bias	Inconsistency	Indirectness	Imprecision	Publication bias	Study event rates (%)		Quality of evidence
						RT alone	SMLI + RT	
ORR (Li, 2021)	Serious^a^	No	No	No	Serious^d^	1,432/1907 (75.1%)	1817/1962 (92.6%)	Low
ADE (Ye et al., 2021)	Serious^a^	No	No	No	No	22/639 (3.4%)	34/640 (5.3%)	Moderate
NIHSS (Li, 2021)	Serious^a^	No^b^	No	No	No	1907	1962	Moderate
BI (Gao et al., 2015)	Serious^a^	No^b^	No	No	Serious^d^	955	963	Low
CRP (Xue et al., 2019)	Serious^a^	No^b^	No	Serious^c^	No	170	170	Low
PV (Zhou et al., 2020)	Serious^a^	No^b^	No	No	No	318	325	Moderate
WBHSV (Li et al., 2020)	Serious^a^	No^b^	No	No	Serious^d^	418	466	Low
WBLSV (Li et al., 2020)	Serious^a^	No^b^	No	No	No	418	466	Moderate
FIB (Li et al., 2020)	Serious^a^	No^b^	No	No	No	337	345	Moderate
HCT (Xue et al., 2019)	Serious^a^	No^b^	No	No	No	204	252	Moderate
PAR (Wang et al., 2017a)	Serious^a^	No^b^	No	No	No	420	475	Moderate

Note:

^a^ Most trialshad unclear risk and the trials were no high risk, but the result had good robustness. The evidence was rated down by only one level.

^b^ Heterogeneity presented in them, and the results had good robustness. Not rated down.

^c^ Thetotalsample size is less than 400 cases. Therefore, the evidence was rated down by one level.

^d^ Publication bias was detected, but the result had good robustness. The evidence was rated down by one level.

Abbreviations: RCTs: randomized controlled trials; SMLI: salvia miltiorrhiza and ligustrazine injection; RT: regular treatments; ORR: objective response rate; ADE: adverse events; NIHSS: national institutes of health stroke scale; BI: barthel index; CRP: C-reactive protein; PV: plasma viscosity; WBHSV: Whole blood high-shear viscosity; WBLSV: Whole blood low-shear viscosity; FIB: fibrinogen; HCT: hematocrit; PAR: platelet aggregation rate.

## 4 Discussion

ACI is one of the common cerebrovascular diseases, and is a major cause of death and disability with an estimated 77 million people suffering from ACI by 2030 (Liu H. et al., 2019). Many scholars hold the opinion that adjuvant therapy, such as traditional Chinese medicine, will provide benefit for patients with ACI (Lyu et al., 2019; Liu H. et al., 2019; Asadi et al., 2015). SML is a traditional Chinese medicine preparation that has been clinically used as an effective adjuvant to reduce brain injury and promote functional recovery (Zhang et al., 2016; Deng et al., 2021; MEIm et al., 2019). Despite statistical analysis of published literatures, there is still limited comprehensive and systematic evaluation of SML in the treatment of ACI. In this analysis, we conducted extensive online searches based on strict inclusion and exclusion criteria, providing an internationally accessible systematic review of the clinical efficacy and safety of SML in treating ACI.

The meta-analysis was conducted on 38 articles (Dai and Wu, 2018; Guo and Guan, 2018; Guo, 2015; Han, 2021; Huang SG. et al., 2016; Ji, 2022; Jiang and Xia, 2019; Lan et al., 2015; Li CL. et al., 2016; Li DQ., 2018; Li, 2021; Li L., 2018; Li and Liu, 2017; Li et al., 2017; Li TD. et al., 2016; Li ZL. et al., 2016; Liu, 2017; Liu, 2018; Liu, 2014; Mamuti, 2017; Qu and Liu, 2016; Song, 2018; Sun, 2017; Tan, 2016; Tan, 2019; Wan et al., 2015; Wang and Wu, 2018; Wang and Zhang, 2016; Xu B. et al., 2021; Xu, 2017; Xu Z. et al., 2021; Yan, 2016; Yang et al., 2010; Yu et al., 2019; Zhang and Li, 2013; Zhang, 2019; Zhang and Xiao, 2020; Zhao and Zhou, 2020) to evaluate the ORR. Compared with conventional treatment alone, the ORR of SML combined with conventional treatment is significantly higher. The combination therapy also significantly improved ADL and the neurological status of ACI patients. CRP is an important indicator in the prediction, prevention and prognosis of ACI (Zhou et al., 2020). Our analysis results indicated that after conventional treatment and combined treatment with SML, the CRP levels of patients significantly decreased. The patient’s hemorheological indicators also showed significant improvement. These results indicated that SML can protect ACI from damage, which may be related to its role in regulating blood viscosity. In order to further eliminate the influence of certain variables on the clinical efficacy of SML in treating ACI, this study conducted a subgroup analysis to determine the effects of different manufacturers of SML and treatment time on ORR and NIHSS. The analysis results showed that the therapeutic effect of SML seems to be unaffected by these variables. However, these analyses involve a limited number of studies and insufficient sample sizes, which may lead to inadequate evaluation. Therefore, these results need to be validated through new evidence and further research.

Safety is one of the key factors affecting the clinical application and further development of drugs. Among the included studies, ten studies (Guo, 2015; Ji, 2022; Lan et al., 2015; Li, 2021; Li ZL. et al., 2016; Liu, 2014; Song, 2018; Sun, 2017; Wang and Wu, 2018; Wang and Zhang, 2016) reported adverse events, and three studies (Huang SG. et al., 2016; Tan, 2016; Zhao and Zhou, 2020) reported no adverse reactions in the two groups. The most common side effects during SML therapy were rash, headache, diarrhea, nausea and vomiting. These side effects are not serious, and will disappear after drug withdrawal or symptomatic treatment. However, the combined use of drugs results in incomplete research information and low methodological quality, and its safety needs further research and clarification.

Although we conducted a systematic analysis, our analysis has some limitations. Firstly, the data we have obtained now were not comprehensive. All included trials were conducted in China. SML, as an important herbal preparation, is mainly used in China, which may inevitably lead to regional deviations and affect the clinical application of SML in the world. This regional bias could limit the generalizability of the findings to other populations. Secondly, current research has detected publication bias in ORR, NIHSS, and adverse events, which may be attributed to some authors tending to publish articles with positive outcomes to editors. Additionally, there is significant heterogeneity in some analyses, particularly in the NIHSS scores (*I* ^*2*^ = 99%). Despite subgroup analysis were conducted, the heterogeneity still exists, which could undermine the reliability of the meta-analysis results. These critical issues are all needed to be addressed in future research in order to avoid overestimating the effectiveness and safety of the treatment. Finally, different trials use different outcome measures to evaluate treatment efficacy, thereby reducing the size of statistical samples, which makes summarizing results on the same scale challenging. Given the identified issues with heterogeneity and publication bias, further prospective, high-quality and multicenter clinical trials are needed to confirm these results.

## 5 Conclusion

In summary, this meta-analysis indicated that SML combined with conventional therapy was effective for ACI patients. The clinical application of SML not only significantly improved the ORR of conventional treatment, but also effectively improved the blood viscosity of ACI patients. However, due to the increased risk and bias of some included low-quality trials, the clinical efficacy and safety of SML-mediated treatment for ACI still requires rigorous methodological trials to validate.

## Data Availability

The raw data supporting the conclusions of this article will be made available by the authors, without undue reservation.
