# Supplementary figures and images for: Safety and effectiveness of Salvia miltiorrhiza and ligustrazine injection for acute cerebral infarction in Chinese population: a PRISMA-compliant meta-analysis
Source: Front Pharmacol. 2024 Dec 2;15:1425053. doi: 10.3389/fphar.2024.1425053 (PMC11646771; doi:10.3389/fphar.2024.1425053)

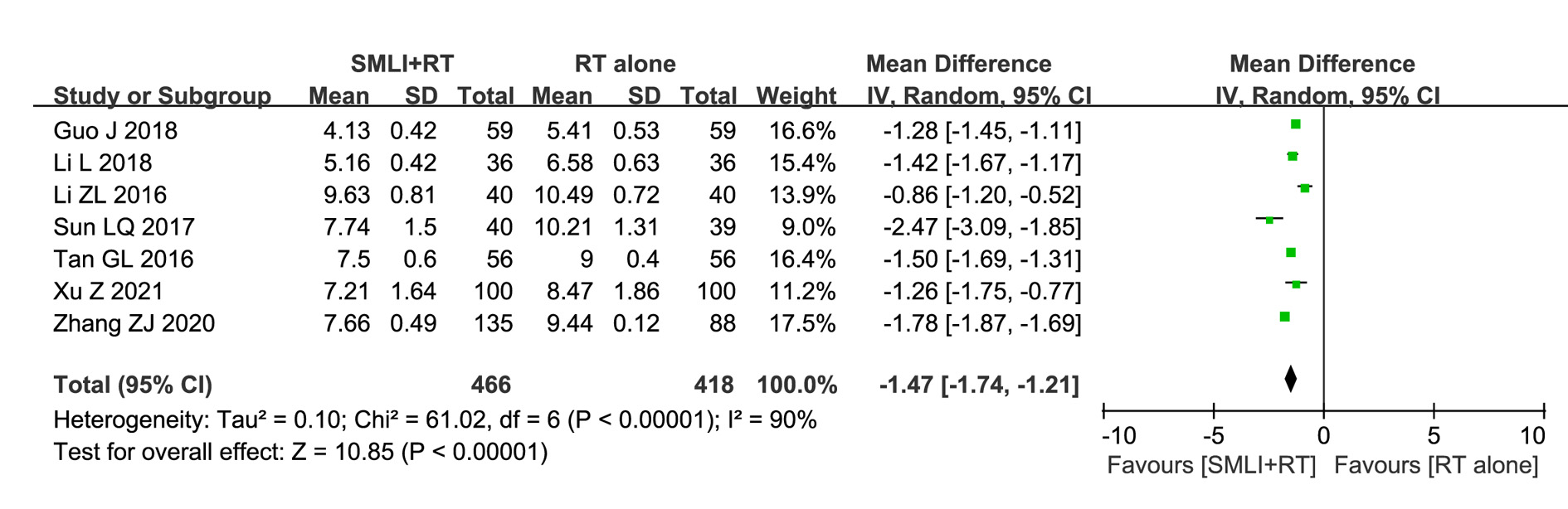

Supplement: Supplementary file 1 [file Image3.JPEG]

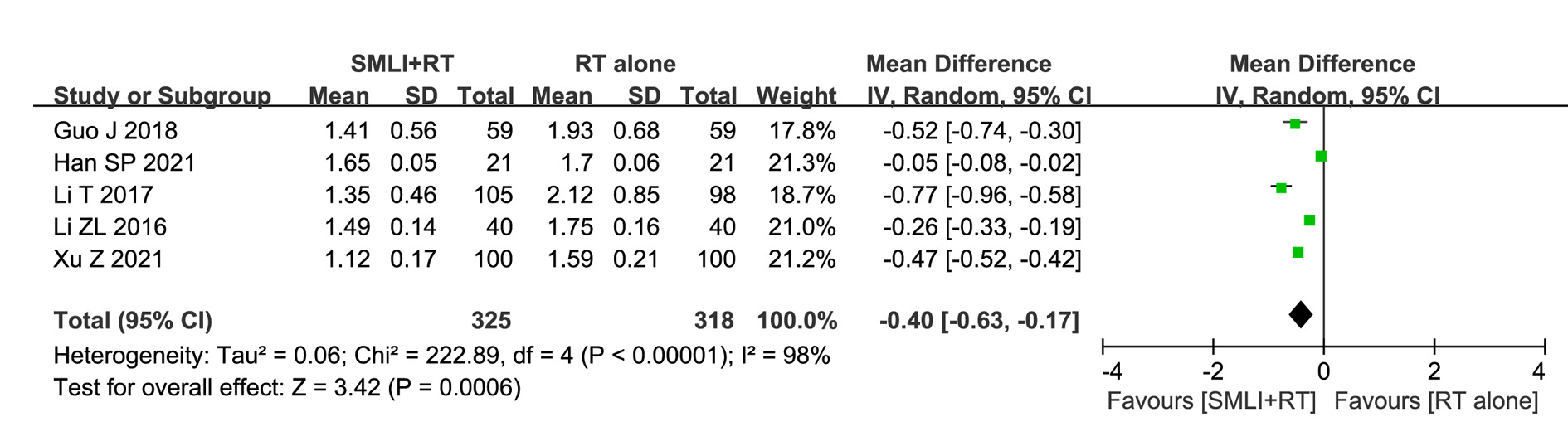

Supplement: Supplementary file 2 [file Image1.JPEG]

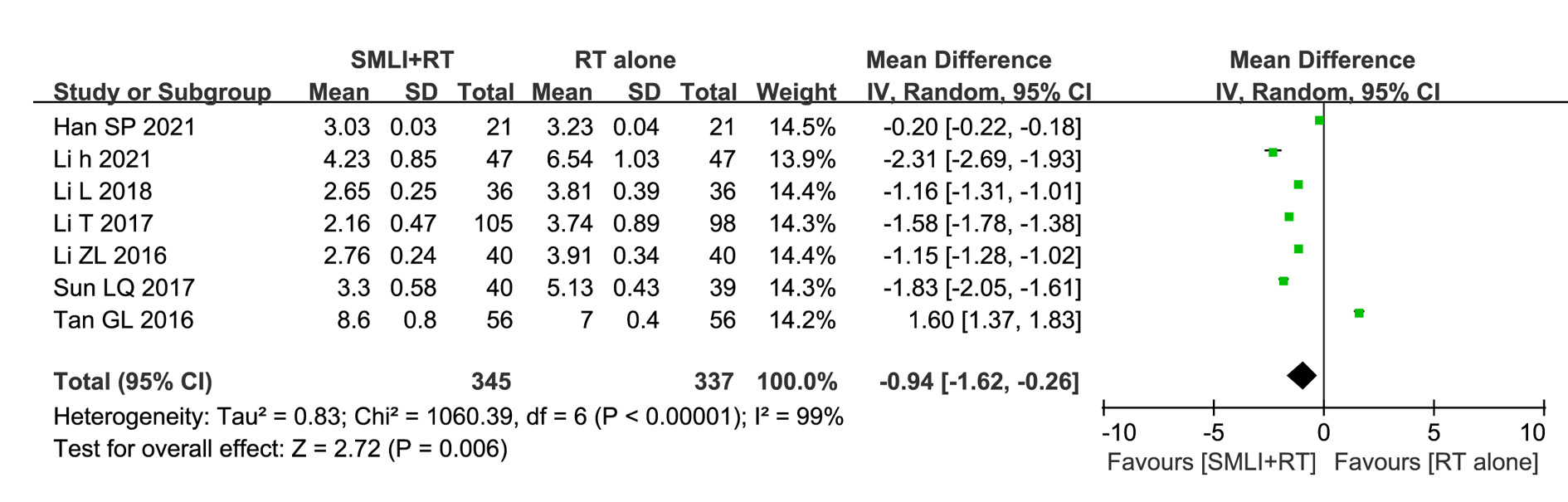

Supplement: Supplementary file 3 [file Image4.JPEG]

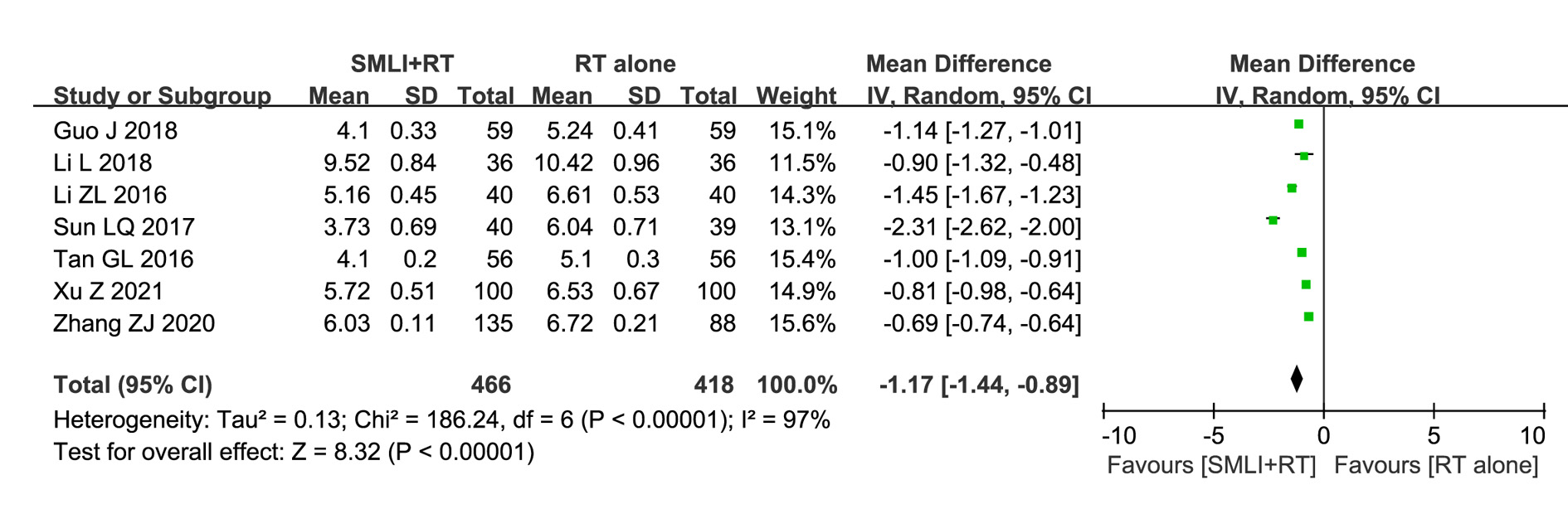

Supplement: Supplementary file 4 [file Image2.JPEG]

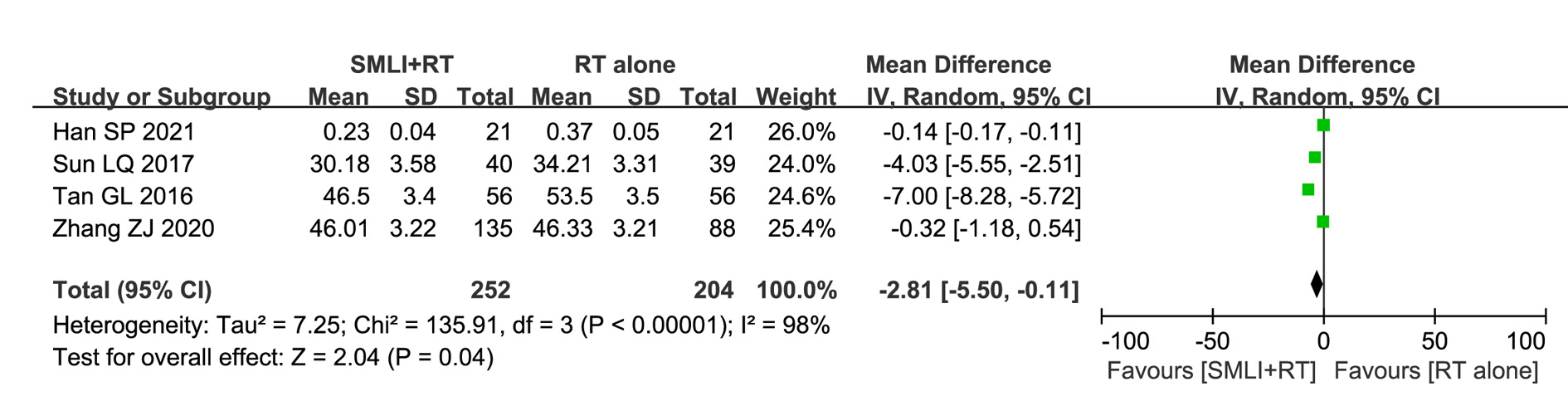

Supplement: Supplementary file 5 [file Image5.JPEG]

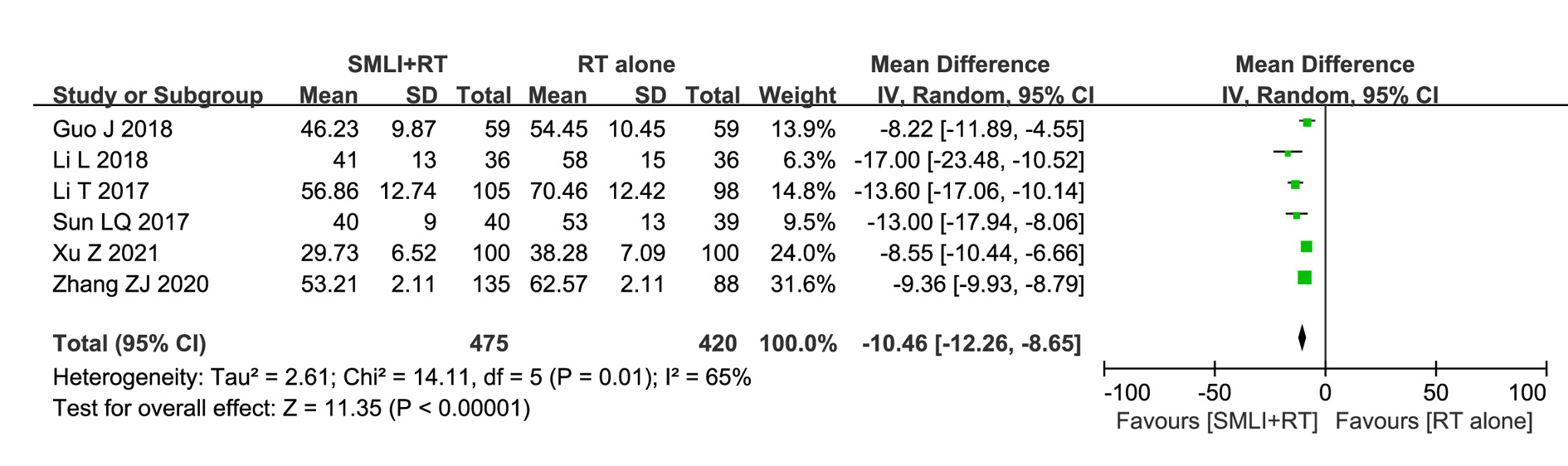

Supplement: Supplementary file 8 [file Image6.JPEG]
