# Supplementary material for: Safety and effectiveness of Salvia miltiorrhiza and ligustrazine injection for acute cerebral infarction in Chinese population: a PRISMA-compliant meta-analysis
Source: Front Pharmacol. 2024 Dec 2;15:1425053. doi: 10.3389/fphar.2024.1425053 (PMC11646771; doi:10.3389/fphar.2024.1425053)
Supplement: Supplementary file 6 [file Table1.doc]

**Supplement Table 1.** Searching strategy for electronic databases

| **Data base** | **Search Strategy** |
| --- | --- |
| **English database:** Cochrane Library, Web of Science, Embase, Medline and PubMed. | **#1.** “cerebral infarction” or “acute cerebral infarction” or “cerebral ischemia” or “brain infarction” or “acute brain infarction” or “infarction of the brain” or “brain ischemia” or “cerebrovascular disorders” or “stroke” or “ischemic stroke” or “intracranial arterial diseases” or “carotid artery diseases” or “intracranial embolism” or “intracranial arterial diseases” or “ischemic cerebrovascular disease” or “ACI” [Title/Abstract].  **#2.** cerebral infarction [MeSH].  **#3. #1** or **#2**.  **#4.** “Salviae Miltiorrhizae and Ligustrazine” or “Radix Salivae Miltiorrhizae Ligustrazine injection” or “Salvia Miltiorrhiza Ligustrazine injection” or “Salviae Miltiorrhizae and Ligustrazine Hydrochloride injection” [Title/Abstract].  **#5.** **#3** and **#4**  **#6.** limit **#5** to human  **#7.** limit **#6** to (Randomized controlled trial)  **#8.** limit #7 to yr = "- May 2023" |
| **Chinese database:** Chinese Scientific Journal Database (VIP), Wanfang database, Chinese Biological Medicine Database (CBM) and China National Knowledge Infrastructure (CNKI). | **#1.** “naogengsi” or “naogengsai” or “jixing naogengsi” or “jixing naogengsai” or **“**cuzhong” or “naocuzhong” or “zhongfeng” or “naozhongfeng” or “quexuexingnaoxueguanbing” or “quexuexingcuzhong” or “quexuexingnaocuzhong” or “quexuexingzhongfeng” or “quexuexingnaozhongfeng” [Title/Keywords].  **#2.** “Danshen Chuanqiongqin” or “Danshen Chuanqiongqin injection” [Title/Keywords].  **#3. #1** and **#2**.  **#4.** limit **#4** to human  **#5.** limit **#5** to (Randomized controlled trial)  **#6.** limit **#6** to yr = "- May 2023" |
