# Supplementary material for: Safety and effectiveness of Salvia miltiorrhiza and ligustrazine injection for acute cerebral infarction in Chinese population: a PRISMA-compliant meta-analysis
Source: Front Pharmacol. 2024 Dec 2;15:1425053. doi: 10.3389/fphar.2024.1425053 (PMC11646771; doi:10.3389/fphar.2024.1425053)
Supplement: Supplementary file 7 [file Table3.docx]

**Supplement Table 3.** Information of the hospitals conducting the relevant research

| **Included studies** | **Hospitals** |
| --- | --- |
| Dai XK 2018 | The Second People's Hospital of Banan District of Chongqing |
| Guo J 2018 | The Second Affiliated Hospital of Henan University of Chinese Medicine |
| Guo Y 2015 | Liaoning Province Mental Health Center |
| Han SP 2021 | Yangzhong Traditional Chinese Medicine Hospital |
| Huang SG 2016 | The Sixth People's Hospital of Chengdu |
| Ji DY 2022 | Kazuo County Central Hospital |
| Jiang KY 2019 | The Second People's Hospital of Neijiang |
| Lan Y 2015 | Xiaolan Hospital Affiliated to Southern Medical University |
| Li CL 2016 | North Branch of The People's Hospital of Xinjiang Uygur Autonomous Region |
| Li DQ 2018 | Henan Provincial Hospital of Traditional Chinese Medicine |
| Li h 2021 | Kangping County People's Hospital |
| Li L 2018 | Yicheng County Chinese Medicine Hospital |
| Li SH 2017 | Wenxi County People's Hospital |
| Li T 2017 | Red Cross Hospital Affiliated to Xi'an Jiaotong University |
| Li TD 2016 | Qiannan Prefecture People's Hospital |
| Li ZL 2016 | The Ninth People's Hospital of Tangshan |
| Liu H 2017 | Nanjing Branch of The Long March Hospital Affiliated to Second Military Medical University |
| Liu JX 2018 | The Fifth People's Hospital of Hengshui |
| Liu M 2014 | The Sixth People's Hospital of Chengdu |
| Mamuti A 2017 | The People's Hospital of Hotan District of Xinjiang |
| Qu J 2016 | The Northern Hospital of Xi'an |
| Song HY 2018 | Hejin People's Hospital |
| Sun LQ 2017 | The People's Hospital Of Anyang City |
| Tan GL 2016 | Affiliated Hospital of Youjiang Medical University for Nationalities |
| Tan HY 2019 | Longmen County People's Hospital |
| Wan J 2015 | Nanjing Branch of Jiangsu Provincial Armed Police Corps Hospital |
| Wang LN 2018 | The Ninth People's Hospital of Xi'an |
| Wang XM 2016 | Xuzhou Tongshan District Traditional Chinese Medicine Hospital |
| Xu B 2021 | Jinhua Traditional Chinese Medicine Hospital |
| Xu HJ 2017 | Xixia County Traditional Chinese Medicine Hospital |
| Xu Z 2021 | The Fourth Affiliated Hospital of Liaoning University of Traditional Chinese Medicine |
| Yan SJ 2016 | Qianjiang Central Hospital |
| Yang ZY 2010 | The First People's Hospital of Kunming |
| Yu J 2019 | The Second People’s Hospital of Wuhu |
| Zhang L 2013 | Beidaihe Sanatorium of Beijing Military Region |
| Zhang QY 2019 | Qinghe County Central Hospital |
| Zhang ZJ 2020 | Chaoyang Kangning Hospital |
| Zhao JQ 2020 | Baoji Hospital Affiliated to Xi'an Medical University |
