# Supplementary material for: Safety and effectiveness of Salvia miltiorrhiza and ligustrazine injection for acute cerebral infarction in Chinese population: a PRISMA-compliant meta-analysis
Source: Front Pharmacol. 2024 Dec 2;15:1425053. doi: 10.3389/fphar.2024.1425053 (PMC11646771; doi:10.3389/fphar.2024.1425053)
Supplement: Supplementary file 9 [file Table2.doc]

**Supplementary Table 2-1.** The results of sensitivity analysis for overall response rate.

| **Study omitted** | **Estimate** | **[95% Conf. interval]** | |
| --- | --- | --- | --- |
|  |  | Lower CI limit | Upper CI limit |
| Dai XK 2018 | 1.2318244 | 1.1966016 | 1.2680839 |
| Guo J 2018 | 1.2322156 | 1.1969687 | 1.2685007 |
| Guo Y 2015 | 1.2336848 | 1.1982366 | 1.2701818 |
| Han SP 2021 | 1.23031 | 1.1954273 | 1.2662107 |
| Huang SG 2016 | 1.2277154 | 1.1928968 | 1.2635502 |
| Ji DY 2022 | 1.2310652 | 1.1958176 | 1.2673516 |
| Jiang KY 2019 | 1.2273961 | 1.1925892 | 1.263219 |
| Lan Y 2015 | 1.2313346 | 1.1962357 | 1.2674633 |
| Li CL 2016 | 1.2324623 | 1.1966616 | 1.269334 |
| Li DQ 2018 | 1.2314976 | 1.1963272 | 1.267702 |
| Li h 2021 | 1.2314453 | 1.1963016 | 1.2676214 |
| Li L 2018 | 1.2306846 | 1.1956871 | 1.2667067 |
| Li SH 2017 | 1.2290603 | 1.1942184 | 1.2649187 |
| Li T 2017 | 1.2383354 | 1.2018926 | 1.2758833 |
| Li TD 2016 | 1.2288991 | 1.1939671 | 1.2648531 |
| Li ZL 2016 | 1.2306303 | 1.1955255 | 1.2667657 |
| Liu H 2017 | 1.2316608 | 1.1964157 | 1.2679445 |
| Liu JX 2018 | 1.2335658 | 1.1985543 | 1.2696 |
| Liu M 2014 | 1.2312865 | 1.1962035 | 1.2673986 |
| Mamuti A 2017 | 1.2310091 | 1.1959611 | 1.2670842 |
| Qu J 2016 | 1.2302972 | 1.1950306 | 1.2666045 |
| Song HY 2018 | 1.2307385 | 1.1956332 | 1.2668746 |
| Sun LQ 2017 | 1.2309332 | 1.1959265 | 1.2669644 |
| Tan GL 2016 | 1.2338047 | 1.1983629 | 1.2702947 |
| Tan HY 2019 | 1.2331408 | 1.1977062 | 1.2696239 |
| Wan J 2015 | 1.2304679 | 1.195475 | 1.2664851 |
| Wang LN 2018 | 1.2328783 | 1.1973873 | 1.2694212 |
| Wang XM 2016 | 1.2329271 | 1.1975507 | 1.2693485 |
| Xu B 2021 | 1.2291775 | 1.1942608 | 1.265115 |
| Xu HJ 2017 | 1.2299259 | 1.1949462 | 1.2659297 |
| Xu Z 2021 | 1.2350553 | 1.1992099 | 1.2719721 |
| Yan SJ 2016 | 1.2311155 | 1.196267 | 1.266979 |
| Yang ZY 2010 | 1.2274593 | 1.1927123 | 1.2632186 |
| Yu J 2019 | 1.2323662 | 1.19713 | 1.2686397 |
| Zhang L 2013 | 1.2329271 | 1.1975507 | 1.2693485 |
| Zhang QY 2019 | 1.2312288 | 1.196173 | 1.267312 |
| Zhang ZJ 2020 | 1.2304193 | 1.1945552 | 1.2673601 |
| Zhao JQ 2020 | 1.2319372 | 1.1967167 | 1.2681942 |
| Combined | 1.2313759 | 1.1966338 | 1.2671268 |

**Supplementary Table 2-2.** The results of sensitivity analysis for neurological deficit score.

| **Study omitted** | **Estimate** | **[95% Conf. interval]** | |
| --- | --- | --- | --- |
|  |  | Lower CI limit | Upper CI limit |
| Dai XK 2018 | -4.33586 | -5.15904 | -3.51268 |
| Guo J 2018 | -4.34803 | -5.16888 | -3.52718 |
| Guo Y 2015 | -4.37981 | -5.19706 | -3.56256 |
| Han SP 2021 | -4.33376 | -5.15112 | -3.5164 |
| Huang SG 2016 | -4.35397 | -5.16877 | -3.53917 |
| Ji DY 2022 | -4.33242 | -5.22009 | -3.44475 |
| Jiang KY 2019 | -4.36281 | -5.17743 | -3.54818 |
| Lan Y 2015 | -4.44607 | -5.26274 | -3.6294 |
| Li CL 2016 | -4.27261 | -5.0867 | -3.45853 |
| Li DQ 2018 | -4.34261 | -5.1611 | -3.52412 |
| Li h 2021 | -4.35004 | -5.16805 | -3.53202 |
| Li L 2018 | -4.22722 | -5.03409 | -3.42035 |
| Li SH 2017 | -4.29924 | -5.11336 | -3.48511 |
| Li T 2017 | -4.42185 | -5.24013 | -3.60357 |
| Li TD 2016 | -4.32344 | -5.13772 | -3.50917 |
| Li ZL 2016 | -4.42738 | -5.12903 | -3.72572 |
| Liu H 2017 | -4.29875 | -5.1149 | -3.4826 |
| Liu JX 2018 | -4.19499 | -5.00565 | -3.38434 |
| Liu M 2014 | -4.33973 | -5.15536 | -3.5241 |
| Mamuti A 2017 | -4.40157 | -5.22346 | -3.57968 |
| Qu J 2016 | -4.21832 | -5.02976 | -3.40689 |
| Song HY 2018 | -4.45359 | -5.26767 | -3.63951 |
| Sun LQ 2017 | -4.36656 | -5.18395 | -3.54916 |
| Tan GL 2016 | -4.3662 | -5.18436 | -3.54803 |
| Tan HY 2019 | -4.3443 | -5.16435 | -3.52425 |
| Wan J 2015 | -4.36196 | -5.1816 | -3.54233 |
| Wang LN 2018 | -4.35803 | -5.17635 | -3.53972 |
| Wang XM 2016 | -4.44317 | -5.2607 | -3.62564 |
| Xu B 2021 | -4.308 | -5.12798 | -3.48803 |
| Xu HJ 2017 | -4.37913 | -5.19607 | -3.56219 |
| Xu Z 2021 | -4.42919 | -5.25667 | -3.60172 |
| Yan SJ 2016 | -4.38324 | -5.1998 | -3.56668 |
| Yang ZY 2010 | -4.33085 | -5.14578 | -3.51593 |
| Yu J 2019 | -4.43794 | -5.25478 | -3.6211 |
| Zhang L 2013 | -4.35712 | -5.17251 | -3.54174 |
| Zhang QY 2019 | -4.27992 | -5.09299 | -3.46685 |
| Zhang ZJ 2020 | -4.26362 | -5.05014 | -3.4771 |
| Zhao JQ 2020 | -4.35869 | -5.19026 | -3.52711 |
| Combined | -4.34828 | -5.15343 | -3.54313 |

**Supplementary Table 2-3.** The results of sensitivity analysis for barthel index score.

| **Study omitted** | | **Estimate** | | **[95% Conf. interval]** | | |
| --- | --- | --- | --- | --- | --- | --- |
|  |  |  |  | Lower CI limit | | Upper CI limit |
| Guo J 2018 | 10.2024 | | 7.583097 | | 12.8217 | |
| Han SP 2021 | 10.31911 | | 7.704572 | | 12.93364 | |
| Ji DY 2022 | 9.952531 | | 7.301022 | | 12.60404 | |
| Lan Y 2015 | 10.54486 | | 7.970238 | | 13.11948 | |
| Li CL 2016 | 9.978822 | | 7.37492 | | 12.58272 | |
| Li T 2017 | 10.22022 | | 7.579441 | | 12.86099 | |
| Mamuti A 2017 | 10.53766 | | 7.962179 | | 13.11314 | |
| Qu J 2016 | 10.3766 | | 7.580969 | | 13.17224 | |
| Sun LQ 2017 | 9.98372 | | 7.349207 | | 12.61823 | |
| Tan GL 2016 | 9.203313 | | 6.735771 | | 11.67086 | |
| Tan HY 2019 | 10.61851 | | 8.069813 | | 13.16721 | |
| Wan J 2015 | 10.41445 | | 7.827229 | | 13.00168 | |
| Wang XM 2016 | 10.67658 | | 8.156423 | | 13.19673 | |
| Xu B 2021 | 10.43714 | | 7.839915 | | 13.03437 | |
| Xu Z 2021 | 10.38347 | | 7.764226 | | 13.00272 | |
| Yan SJ 2017 | 10.19768 | | 7.623009 | | 12.77235 | |
| Yu J 2019 | 10.35594 | | 7.7596 | | 12.95228 | |
| Zhao JQ 2020 | 10.43056 | | 7.817557 | | 13.04357 | |
| Combined | 10.26877 | | 7.745528 | | 12.79201 | |

**Supplementary Table 2-4.** The results of sensitivity analysis for Serum C-reactive protein.

| **Study omitted** | | **Estimate** | | **[95% Conf. interval]** | | |
| --- | --- | --- | --- | --- | --- | --- |
|  |  |  |  | Lower CI limit | | Upper CI limit |
| Ji DY 2022 | -5.10605 | | -7.33849 | | -2.8736 | |
| Li h 2021 | -3.59462 | | -4.47082 | | -2.71842 | |
| Li SH 2017 | -4.51569 | | -6.7479 | | -2.28348 | |
| Xu B 2021 | -4.78346 | | -7.74607 | | -1.82084 | |
| Combined | -4.53438 | | -6.4334 | | -2.63535 | |

**Supplementary Table 2-5.** The results of sensitivity analysis for plasma viscosity.

| **Study omitted** | | **Estimate** | | **[95% Conf. interval]** | | |
| --- | --- | --- | --- | --- | --- | --- |
|  |  |  |  | Lower CI limit | | Upper CI limit |
| Guo J 2018 | -0.375517 | | -0.628939 | | -0.122095 | |
| Han SP 2021 | -0.486501 | | -0.663357 | | -0.309645 | |
| Li T 2017 | -0.31548 | | -0.5555 | | -0.07545 | |
| Li ZL 2016 | -0.443177 | | -0.755948 | | -0.130407 | |
| Xu Z 2021 | -0.378955 | | -0.613505 | | -0.144405 | |
| Combined | -0.401202 | | -0.631032 | | -0.171371 | |

**Supplementary Table 2-6.** The results of sensitivity analysis for whole blood high-shear viscosity.

| **Study omitted** | | **Estimate** | | **[95% Conf. interval]** | | |
| --- | --- | --- | --- | --- | --- | --- |
|  |  |  |  | Lower CI limit | | Upper CI limit |
| Guo J 2018 | -1.176369 | | -1.491367 | | -0.86137 | |
| Li L 2018 | -1.205003 | | -1.501843 | | -0.908163 | |
| Li ZL 2016 | -1.1202 | | -1.403978 | | -0.836422 | |
| Sun LQ 2017 | -0.99491 | | -1.21673 | | -0.77308 | |
| Tan GL 2016 | -1.20602 | | -1.5737 | | -0.83834 | |
| Xu Z 2021 | -1.23428 | | -1.55511 | | -0.91344 | |
| Zhang ZJ 2020 | -1.25781 | | -1.55705 | | -0.95856 | |
| Combined | -1.16916 | | -1.444713 | | -0.893606 | |

**Supplementary Table 2-7.** The results of sensitivity analysis for whole blood low-shear viscosity.

| **Study omitted** | | **Estimate** | | **[95% Conf. interval]** | | |
| --- | --- | --- | --- | --- | --- | --- |
|  |  |  |  | Lower CI limit | | Upper CI limit |
| Guo J 2018 | -1.5111 | | -1.80782 | | -1.21437 | |
| Li L 2018 | -1.48439 | | -1.79182 | | -1.17697 | |
| Li ZL 2016 | -1.5634 | | -1.81352 | | -1.31327 | |
| Sun LQ 2017 | -1.37499 | | -1.64213 | | -1.10785 | |
| Tan GL 2016 | -1.47258 | | -1.80173 | | -1.14343 | |
| Xu Z 2021 | -1.4995 | | -1.78463 | | -1.21437 | |
| Zhang ZJ 2020 | -1.39639 | | -1.65058 | | -1.1422 | |
| Combined | -1.47224 | | -1.73815 | | -1.20633 | |

**Supplementary Table 2-8.** The results of sensitivity analysis for content of fibrinogen.

| **Study omitted** | | **Estimate** | | **[95% Conf. interval]** | | |
| --- | --- | --- | --- | --- | --- | --- |
|  |  |  |  | Lower CI limit | | Upper CI limit |
| Han SP 2021 | -1.06827 | | -1.94095 | | -0.19559 | |
| Li h 2021 | -0.72113 | | -1.42629 | | -0.01596 | |
| Li L 2018 | -0.90539 | | -1.69056 | | -0.12023 | |
| Li T 2017 | -0.83435 | | -1.55833 | | -0.11037 | |
| Li ZL 2016 | -0.90744 | | -1.72205 | | -0.09284 | |
| Sun LQ 2017 | -0.79283 | | -1.49715 | | -0.08851 | |
| Tan GL 2016 | -1.3622 | | -2.0402 | | -0.6842 | |
| Combined | -0.94126 | | -1.6191 | | -0.26341 | |

**Supplementary Table 2-9.** The results of sensitivity analysis for hematocrit.

| **Study omitted** | | **Estimate** | | **[95% Conf. interval]** | | |
| --- | --- | --- | --- | --- | --- | --- |
|  |  |  |  | Lower CI limit | | Upper CI limit |
| Han SP 2021 | -3.76316 | | -8.01588 | | 0.48956 | |
| Sun LQ 2017 | -2.42038 | | -5.50994 | | 0.669182 | |
| Tan GL 2016 | -1.29244 | | -2.87109 | | 0.286212 | |
| Zhang ZJ 2020 | -3.69199 | | -8.35827 | | 0.974289 | |
| Combined | -2.80727 | | -5.50041 | | -0.11413 | |

**Supplementary Table 2-10.** The results of sensitivity analysis for platelet aggregation rate.

| **Study omitted** | | **Estimate** | | **[95% Conf. interval]** | | |
| --- | --- | --- | --- | --- | --- | --- |
|  |  |  |  | Lower CI limit | | Upper CI limit |
| Guo J 2018 | -10.9492 | | -13.0428 | | -8.85563 | |
| Li L 2018 | -9.87007 | | -11.4139 | | -8.32625 | |
| Li T 2017 | -9.72143 | | -11.359 | | -8.08387 | |
| Sun LQ 2017 | -10.1797 | | -12.0554 | | -8.30399 | |
| Xu Z 2021 | -11.4288 | | -14.0826 | | -8.77506 | |
| Zhang ZJ 2020 | -11.4001 | | -14.395 | | -8.40519 | |
| Combined | -10.4583 | | -12.2635 | | -8.65306 | |

**Supplementary Table 2-11.** The results of sensitivity analysis for adverse events.

| **Study omitted** | | **Estimate** | | **[95% Conf. interval]** | | |
| --- | --- | --- | --- | --- | --- | --- |
|  |  |  |  | Lower CI limit | | Upper CI limit |
| Guo Y 2015 | 1.41978 | | 0.854245 | | 2.359715 | |
| Huang SG 2016 | 1.494211 | | 0.907863 | | 2.459253 | |
| Ji DY 2022 | 1.990349 | | 1.135541 | | 3.488636 | |
| Lan Y 2015 | 1.377318 | | 0.826179 | | 2.296119 | |
| Li h 2021 | 1.493685 | | 0.888071 | | 2.512295 | |
| Li ZL 2016 | 1.41978 | | 0.854204 | | 2.359828 | |
| Liu M 2014 | 1.515651 | | 0.912878 | | 2.516435 | |
| Song HY 2018 | 1.466445 | | 0.831891 | | 2.585028 | |
| Sun LQ 2017 | 1.55 | | 0.8991 | | 2.672117 | |
| Tan GL 2016 | 1.494211 | | 0.907863 | | 2.459253 | |
| Wang LN 2018 | 1.564642 | | 0.923575 | | 2.650683 | |
| Wang XM 2016 | 1.292395 | | 0.77035 | | 2.168214 | |
| Zhao JQ 2020 | 1.494211 | | 0.907863 | | 2.459253 | |
| Combined | 1.494211 | | 0.907863 | | 2.459253 | |
